# Arteriovenous fistula in the aortic arch: a case report involving 3D models for surgical planning

**DOI:** 10.1093/ehjcr/ytaf362

**Published:** 2025-07-30

**Authors:** Clara Isabel Perez, Lore Lakunza, Alberto Saenz, Roberto Voces, Ignacio Gallo

**Affiliations:** Cardiac Surgery, Policlínica Gipuzkoa, Pº de Miramon 174, 20014 Donostia, Spain; Multidisciplinary 3D Printing Platform, Biogipuzkoa Healthcare Investigation Institute, Pº Dr Begiristain, 20014 Donostia, Spain; Cardiac Surgery, Policlínica Gipuzkoa, Pº de Miramon 174, 20014 Donostia, Spain; Cardiac Surgery, Salamanca University Hospital, Pº de San Vicente 182, 37007 Salamanca, Spain; Cardiac Surgery, Policlínica Gipuzkoa, Pº de Miramon 174, 20014 Donostia, Spain

**Keywords:** 3D modelling, Traumatic arteriovenous fistula, Fistula aortic arch, Aorta case report

## Abstract

**Background:**

Traumatic arteriovenous fistulas involving the thoracic aorta are rare. Their closure remains a challenge for the surgeon due to the vascular changes involved. We aim to highlight the benefits of 3D models and virtual three-dimensional reconstruction when used as a complementary tool in surgical planning.

**Case summary:**

We present the case of a 38-year-old male patient with a history trauma from a localized bombing, resulting in shrapnel impact to his thorax. He came for surgical treatment due to a diagnosis of arteriovenous fistula at the level of the aortic arch. For surgical planning, two 3D models (with FDM and PolyJet technology) were made to assess the anatomical relationship between the aorta, the left brachiocephalic vein, the fistulous tract and the sternum. The patient was successfully operated on.

**Discussion:**

Surgical repair of thoracic fistulas is highly complex, requiring a multi-disciplinary approach and advanced imaging for accurate diagnosis and planning. Procedures are often performed under cardiopulmonary bypass and hypothermia to enhance safety, especially in challenging scenarios. These fistulas frequently cause vascular overload and degenerative venous changes, increasing the risk of haemorrhage and complicating dissection. 3D technology significantly improves surgical planning by enabling accurate fistula localization, guiding sternotomy, simulating procedures, and mapping vascular anomalies. PolyJet models, in particular, provide superior anatomical insight. Thus, 3D modelling serves as a complementary tool that provides life-size, detailed anatomical information of the patient, facilitating better pre-operative surgical planning, and carrying the potential to reduce operative risks.

Learning points

- Surgical treatment of arteriovenous fistula involving the thoracic aorta presents significant technical challenges.
- 3D modelling serves as a valuable complementary tool, enhancing the understanding of rare pathologies and enabling optimal surgical planning.

## Background

Traumatic arteriovenous fistulas in the thoracic aorta are rare, as penetrating injuries in this region are usually fatal. Understanding the anatomy and the relationship between the aorta and surrounding anatomical structures is crucial before surgery.^1^ The hyperflux caused by such a fistula can lead to vascular irregularities, arterial steal syndrome, heart failure, and left-right shunts.

3D technology offers a virtual, tangible and full-scale model of the patient’s pathology, enabling personalized and precise surgical planning.

We present a case study where a patient experiences an arteriovenous fistula between the ascending aorta and left brachiocephalic vein (LBV) and is successfully treated with the aid of 3D technology for surgical planning.

## Summary figure

**Figure:**
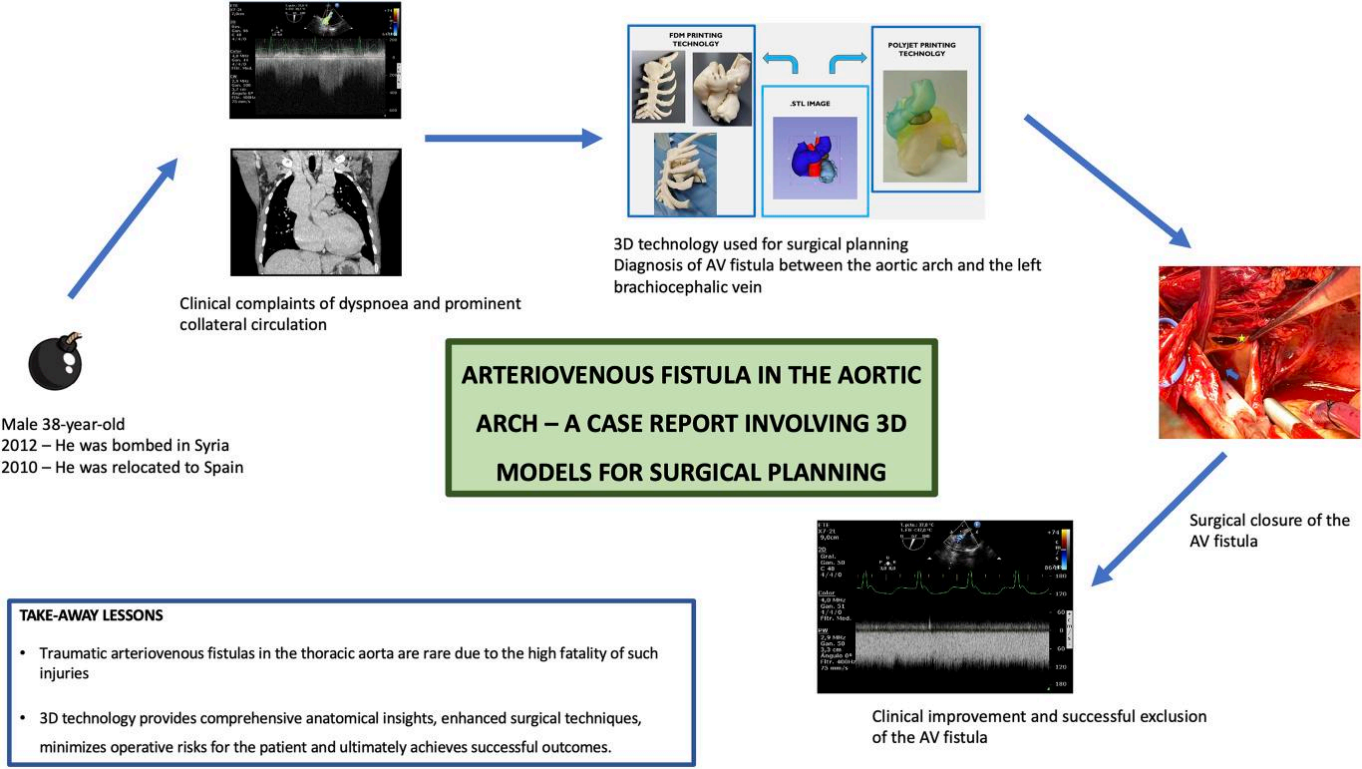


## Case summary

A 38-year-old male patient Syrian refugee presented for cardiac evaluation due to dyspnoea on minimal exertion. In 2012, he sustained shrapnel injuries and chest trauma during a localized bombing in his country, leading to prolonged hospitalization. Details of his hospitalization could not be obtained. He reported increased collateral thoracic circulation since the injury. A previous echocardiogram showed dilated heart chambers, moderate to severe tricuspid regurgitation and severe pulmonary hypertension. After spending 10 years in a refugee camp, he was relocated to Spain in 2022.

He was diagnosed with hypertension and started on medical treatment. Medical examination revealed a panfocal thrill over the precordium and good distal pulses. Echocardiography showed chamber dilation, moderate tricuspid insufficiency, PSP 55–60 mmHg, and continuous flow at the aortic arch, suggestive of a fistula.

**Figure 1:**
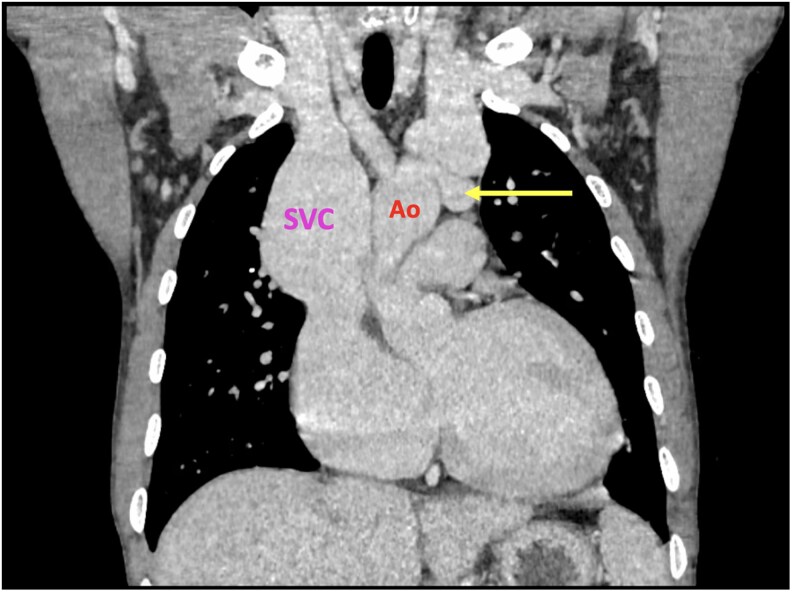
Computerized tomography (CT) coronary view. The yellow arrow indicates the arteriovenous fistula. SVC, superior vena cava; Ao, aorta.

On this basis, a computerized tomography (CT) was performed; it confirmed a 7 mm fistula between the aortic arch and the LBV, with significant venous dilatation (*Figure 1*). Aortography supported the fistula diagnosis, showing an oximetric jump.

Due to the complex anatomical relationship with the supra-aortic vessels, an endovascular approach was ruled out. The patient was referred for surgical closure of the fistula. For further understanding of the pathology and surgical planning, 3D technology was utilized.

CT images were converted to standard triangle language or standard tessellation language files for 3D virtual reconstruction, producing models that highlighted critical anatomical relationships between: The sternum, clavicle, rib bones, pulmonary vein, aorta, vena cava with the LBV and its pathologic fistula.

**Figure 2:**
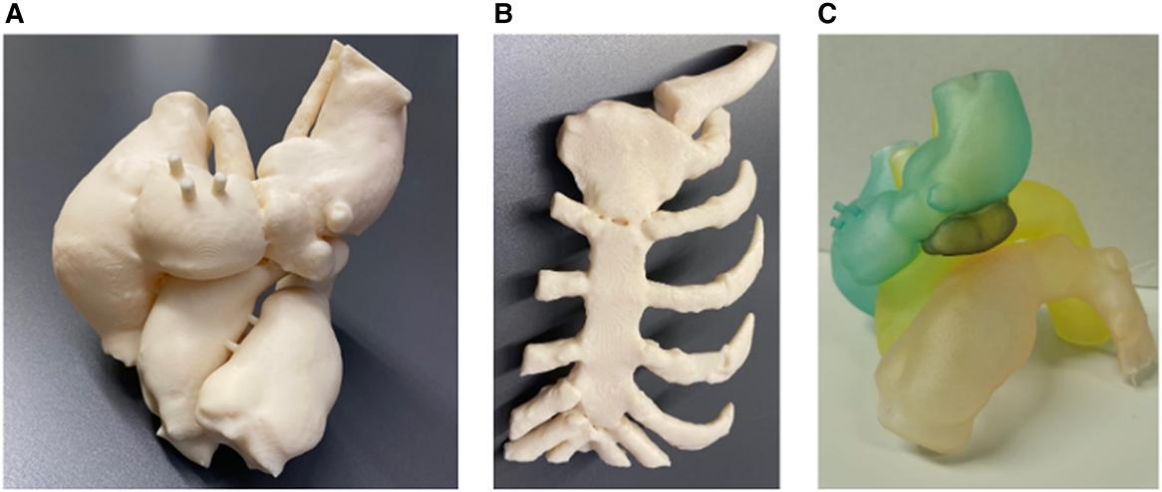
3D printing models. (*A*) Fused deposition modelling (FDM) model of the superior vena cava, aorta, innominate vein, and pulmonary artery. (*B*) FDM model of the sternum, the rib bones, and the left clavicle. (*C*) PolyJet model of the great vessels and the fistulous tract.

**Figure 3:**
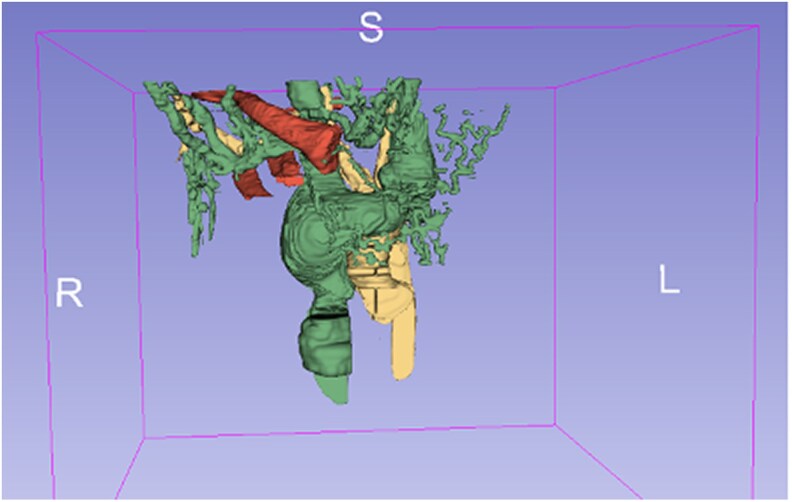
3D virtual reconstruction of the vascular network. Venous vessels represented in green, arterial vessels in yellow. The clavicle is represented in red.

Pre-operative 3D printed models [using FDM (Fused Deposition Modelling) and PolyJect technology] facilitated surgical planning, showing the fistula’s connection to the aorta and the surrounding vascular structures (*Figure 2A and B*). PolyJet technology provided flexible, detailed models for simulating surgical techniques (*Figure 2C*). Virtual reconstruction mapped the hyper developed vascular network, aiding in the identification of the veins and surgical approaches (*Figure 3*).

A week before surgery, the patient came in for another pre-operative visit. On this occasion, the interview was conducted using the 3D models, which allowed the patient and the family to experience a better understanding of the pathology and the surgical approach that was going to be performed.

During surgery, the patient was positioned supine and underwent general anaesthesia. Anatomical landmarks were marked and the area most accessible for cannulation was identified from the right subclavian artery. Cardiopulmonary bypass with core cooling was initiated and a median sternotomy using an oscillating saw was performed, to avoid damage to the LBV. Careful dissection was implemented to the veins due to their particular position and being at high risk for bleeding as identified by the virtual reconstruction. A thrill was observed on the LBV.

Once the patient reached 25°, the aorta above the coronary arteries was cross-clamped and cardioplegia was infused through the aorta and the heart was put under cardiac arrest. Subsequently, complete circulatory interruption with cerebral perfusion was initiated. The ascending aorta was incised with a curved line longitudinally towards the arch.

**Figure 4:**
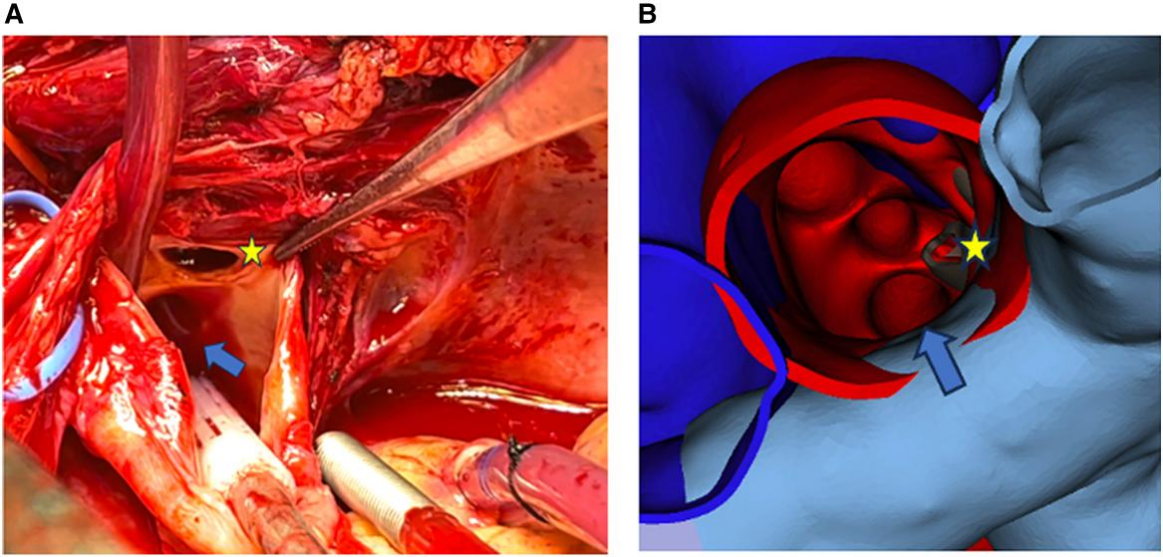
Aortic orifices of left subclavian artery (blue arrow) and fistula (yellow star) through the aorta. (*A*) Surgical view. (*B*) 3D virtual reconstruction view.

The aortic orifice of the fistula was identified and verified against the 3D model (*Figure 4*) and closed with an interrupted suture. The procedure involved 13 min of cerebral perfusion, after which the aorta was unclamped and the heart resumed sinus rhythm. When the patient reached normal temperature and exhibited good hemodynamic, cardiopulmonary bypass was discontinued.

**Figure 5:**
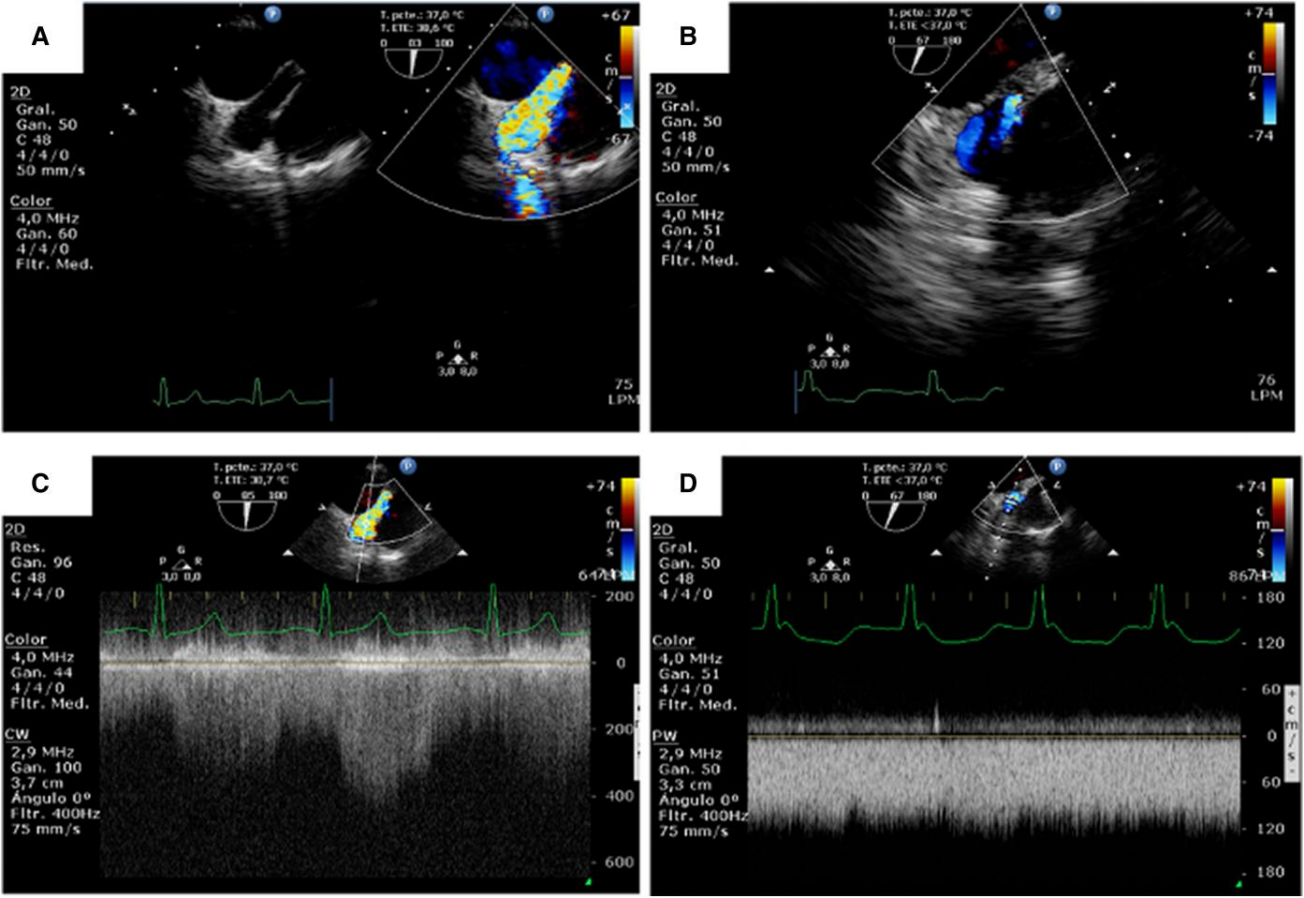
Echo Doppler of the fistulous tract pre- (*A*, *C*) and post-surgery (*B*, *D*). (*A*) Turbulent flow in the fistulous tract. (*C*) Pulsatile flow inside the fistulous tract. (*B*) Absence of the turbulent flow in the fistulous tract. (*D*) Laminar flow inside the fistulous tract.

A post-operative transoesophageal echocardiogram (TEE) confirmed the absence of turbulent blood flow (*Figure 5*). The patient had an uneventful recovery and was extubated after 6 h. He was discharged 7 days post-operatively. Follow-up post-op assessment showed no dyspnoea and reduced thoracic collateral circulation.

## Discussion

Traumatic fistulas located in the thoracic area are a rare complication with a high risk for closure, due to their relatively inaccessible location and the hemodynamic changes resulting from the fistula’s pathophysiology.^2^ Managing such complex cases requires a multi-disciplinary approach, often involving advanced imaging techniques and careful surgical planning.

Historically, successful surgical repairs of these types of fistulas have been reported using various techniques, with or without cardiopulmonary bypass. Recent literature suggests that cardiopulmonary bypass and hypothermia provide a safety mechanism for approaching this pathology, especially when the diagnosis is uncertain, to minimize complications.^3,4^

It is well known that identification of the fistula orifices and tracts is complex and critical. Therefore, modern diagnostic techniques such as TEE,^5^ CT, or MRI are crucial for precise localization.

Arteriovenous fistulas cause vascular overload, leading to degenerative vascular changes, particularly in the venous network.^6^ This complicates the surgical field and increases the risk of haemorrhaging, highlighting the need for meticulous planning and execution.

Reddi *et al.*^3^ identified two stages of great importance during this type of surgery. First, during the retraction of the sternum after median sternotomy and secondly, during the dissection around the fistula.

Surgical strategies for managing aorto-venous fistulas, particularly those involving the thoracic aorta, focus on reconstructing the arterial vessel and preventing cerebral ischaemia.^7^

3D technology has emerged as a valuable tool in surgical planning, providing detailed and accurate anatomical models that enhance the understanding of complex structures and facilitate better surgical outcomes.^8,9^ Benefits were noted in the following areas:

1. Sternotomy planning: the anatomical models helped us to avoid intraoperative vessel rupture by clearly demonstrating the proximity of the LBV and the fistulous tract to the sternum and rib bones.
2. Fistula localization: 3D models offered a more accurate representation of the patient’s anatomy compared to conventional images, aiding in the precise localization of the fistula. The Polyjet model was superior than the FDM. One way it is superior is that it allows for the combination of colours, transparent materials and facilitates greater details.^10^
3. Surgical strategy: the flexibility and detail of the PolyJet model allowed to simulate the procedure pre-operatively and plan the surgical approach, ensuring the integrity of the vein by opting for a no-touch technique.
4. Vascular network mapping: virtual 3D reconstructions were invaluable in mapping the venous abnormalities caused by chronic hyperflux, facilitating the dissection and cannulation process.

It goes without saying that for challenging cases, there are different surgical approaches that can be performed. However, we strongly believe that the 3D technology is a revolutionary innovation that can be used as a complementary tool not only in the diagnosis of a patient, but also in the surgical planning, in order to achieve better outcomes reducing operative risks.

## Conclusions

In summary, traumatic arteriovenous fistulas of the ascending aorta are rare, challenging to diagnose and difficult to treat. This necessitates meticulous surgical planning. The use of 3D technology provides comprehensive anatomical insights, enhanced surgical techniques, minimizes operative risks for the patient and ultimately achieves successful outcomes.

## Data Availability

The data underlying this article will be shared on reasonable request to the corresponding author.

## Lead author biography

She graduated and master’s degree in medicine in 2016 from the Universidad Complutense of Madrid. She completed her training as a cardiovascular surgeon in Spain in 2022 at the Cruces University Hospital. Since then, she has been working as a heart surgeon at Policlínica Gipuzkoa. For the last 5 years, she has been doing research in 3D technology. She is currently doing her PhD programme in medicine and surgery at the Autónoma University of Madrid.

## Notes

**Consent:** The authors confirm that witnessed verbal consent for submission and publication of this case report including images and associated text has been obtained from the patient. This has been discussed with the editors.

**Funding:** No funding.
